# A Real‐World Study of Tafasitamab for the Treatment of Relapsed or Refractory Diffuse Large B‐Cell Lymphoma in the United States

**DOI:** 10.1002/jha2.70256

**Published:** 2026-03-26

**Authors:** Kim Saverno, Loretta J. Nastoupil, Bruce Feinberg, John Galvin, Prathamesh Pathak, Theresa Amoloja, Danielle Gentile, Parisa Asgarisabet, Narendranath Epperla

**Affiliations:** ^1^ Incyte Corporation Wilmington Delaware USA; ^2^ Department of Lymphoma/Myeloma University of Texas MD Anderson Cancer Center Houston Texas USA; ^3^ Southwest Oncology CommonSpirit Oncology Mercy Durango Colorado USA; ^4^ Cardinal Health Dublin Ohio USA; ^5^ Division of Hematology The Ohio State University Columbus Ohio USA; ^6^ Division of Hematology and Hematologic Malignancies Huntsman Cancer Institute University of Utah Salt Lake City Utah USA

**Keywords:** CD19, DLBCL, immunotherapy, real‐world, tafasitamab

## Abstract

**Background:**

Tafasitamab, a CD19‐targeting immunotherapy, is approved in combination with lenalidomide for the treatment of adults with relapsed or refractory diffuse large B‐cell lymphoma (R/R DLBCL) ineligible for autologous stem cell transplant. This real‐world study describes patient characteristics, treatment patterns, and outcomes associated with tafasitamab for R/R DLBCL across US practice settings.

**Methods:**

A retrospective physician‐abstracted chart review was conducted among US adults initiating tafasitamab (with/without lenalidomide) for R/R DLBCL as part of routine clinical care. Data were summarized using descriptive statistics. Cox proportional hazards‐based analyses were performed to determine baseline characteristics associated with real‐world progression‐free survival and overall survival.

**Results:**

Twenty‐three physicians (83% community physicians) provided data on 181 patients (64% White; median follow‐up since initiation, 14.7 months) meeting eligibility criteria. Most patients (72%) received tafasitamab as second‐line (2L) therapy. Real‐world overall response rate (95% CI) to tafasitamab was 73.5% (67.0–79.9). Median (95% CI) real‐world progression‐free survival and overall survival from tafasitamab initiation were 11.3 (9.8–13.6) and 24.8 (17.8–NE) months, respectively. In multivariable Cox proportional hazards‐based analyses, tafasitamab use in third‐line to fifth‐line (3L–5L) (vs. 2L), bulky disease (vs. non‐bulky), Ann Arbor Stage III–IV (vs. stage I–II), and increasing Charlson Comorbidity Index scores were determined as factors associated with increased progression risk. In addition, increasing age, tafasitamab in 3L–5L (vs. 2L), bulky disease (vs. non‐bulky), and Eastern Cooperative Oncology Group performance status (ECOG PS) ≥ 2 (vs. < 2) were associated with higher mortality risk.

**Conclusion:**

This analysis of a diverse US population who received tafasitamab for R/R DLBCL predominantly in community practice settings supports the clinical benefit of tafasitamab.

**Trial Registration:**

The authors have confirmed clinical trial registration is not needed for this submission.

## 1 Introduction

Diffuse large B‐cell lymphoma (DLBCL) is the most common subtype of non‐Hodgkin lymphoma (NHL), representing approximately 40% of all NHL cases globally, with an estimated 77,240 new cases in the United States in 2020 and an incidence of 7 per 100,000 in the United States [1, 2, 3, 4].

Approximately 30%–40% of patients with DLBCL have disease that relapses or is refractory to first‐line (1L) treatment [5]. Historically, consolidation therapy with high‐dose chemotherapy (HDT) and autologous stem cell transplant (ASCT) was a preferred second‐line (2L) option for fit patients with chemotherapy‐sensitive disease [6]. In the current era, chimeric antigen receptor T‐cell (CAR‐T) therapy is suggested for eligible patients with relapse within 12 months following 1L, whereas HDT/ASCT is considered for later relapse in young fit patients, should they achieve chemosensitivity [6].

Tafasitamab is a CD19‐targeted immunotherapy indicated in combination with lenalidomide for treatment of adult patients with relapsed/refractory (R/R) DLBCL ineligible for ASCT [7]. Approval by the US Food and Drug Administration on July 31, 2020, was based on findings from the L‐MIND multicenter, open‐label, single‐arm, Phase II trial (NCT02399085). This trial included 80 ASCT‐ineligible patients with R/R DLBCL who received ≥ 1 dose of intravenous tafasitamab (12 mg/kg) co‐administered with oral lenalidomide (25 mg) for up to 12 cycles (28 days each), followed by tafasitamab as monotherapy in patients with stable disease or better until disease progression [8, 9, 10]. In the primary analysis (median follow‐up, 13.2 months), the objective response rate for tafasitamab‐based treatment was 60% (95% confidence interval [CI], 48–71), with 34 patients (43%; 95% CI, 32–54) achieving a complete response (CR) [8].

Although some real‐world studies have evaluated tafasitamab in the United States since its approval [11, 12, 13, 14, 15, 16, 17], studies that report on effectiveness from a sizeable number of patients receiving tafasitamab for R/R DLBCL from both academic and community practices are scarce. One of the largest real‐world studies examining the effectiveness of tafasitamab for R/R DLBCL in the United States included patients from academic centers that were primarily White, and a significant proportion had heavily pre‐treated (3L+) and aggressive disease [13]. A study addressing the lack of diversity in prior real‐world studies, and which includes patients treated with tafasitamab in the 2L setting, will be more reflective of community practice. Hence, we sought to evaluate patient characteristics, treatment patterns, and outcomes associated with tafasitamab for R/R DLBCL across practice settings in the United States as part of routine clinical care.

## 2 Methods

### 2.1 Data Source and Study Design

This real‐world study was a retrospective, observational, multisite, physician‐abstracted medical chart review of adults who received tafasitamab for R/R DLBCL in the United States as a part of routine clinical care. Data were abstracted from medical records of US patients who met pre‐specified eligibility criteria by oncologists from the Cardinal Health Oncology Provider Extended Network (OPEN). OPEN is a geographically diverse, electronic medical record/group purchasing organization–agnostic, community of US physicians across community and academic practice settings. Email invitations were sent to roughly 1150 physicians in OPEN. Physicians voluntarily participated in the study by completing electronic case report forms (eCRFs) on eligible patients. Physicians with more than 10 eligible patients were instructed to first compile a comprehensive list of all eligible patient charts in chronological order, beginning with the earliest qualifying case. From this complete set, up to 10 patients were then randomly selected for data abstraction. This approach was designed to ensure that patient selection followed a standardized process and incorporated randomisation within predefined limits. Physicians provided information about their practice characteristics and abstracted data from medical records related to patient demographics, clinical characteristics, treatment patterns, and outcomes in eCRFs. Initial data collection occurred from February 22, to March 29, 2023. Approximately 10 months later, between December 18, 2023, and January 31, 2024, physicians were asked to provide additional follow‐up data on patients who were still alive at the last follow‐up during initial data collection.

For data related to treatment sequencing, a line of therapy was considered to have ended when the patient discontinued treatment with a regimen; if a drug was added to ongoing treatment, this was considered a new line of therapy, with the exceptions that bridging therapy followed by CAR‐T was classified as a single line of therapy, and salvage chemotherapy followed by HDT/ASCT was classified as a single line of therapy. If a drug within a combination was held or discontinued, this was not considered a new line of therapy.

The study followed all standard research guidelines and received approval and exemption for obtaining patient‐informed consent by a central institutional review board.

### 2.2 Patient Population

Eligible patients received tafasitamab for R/R DLBCL on or after October 21, 2020, and were ≥ 18 years of age at initiation. Concomitant use of lenalidomide was not a requirement for study participation. Patients had ≥ 4 months of follow‐up time since initiation with tafasitamab, with the exception that an otherwise eligible patient who died during the 4‐month follow‐up period was still included. Patients who received tafasitamab as part of an interventional clinical trial were excluded from analysis.

### 2.3 Statistical Analysis

Data from the two data collection periods were merged and summarised using descriptive statistics; time‐to‐event endpoints were summarised using the Kaplan–Meier method. Univariable analyses were conducted to assess potential associations between patient and treatment characteristics, with real‐world progression‐free survival (rwPFS) and overall survival (rwOS) as the endpoints of interest. rwPFS was defined as time from initiation of tafasitamab to physician‐reported disease progression or death from any cause, whichever occurred first. rwOS was defined as the time from tafasitamab initiation to death from any cause. Variables with *p *≤ 0.2 in univariable analyses were included in initial multivariable Cox proportional hazards models. Backward selection with *p *< 0.05 was used to determine which factors, when adjusted for others, were associated with rwPFS and rwOS among patients who had initiated tafasitamab for R/R DLBCL. Best model fit was determined by the Akaike information criterion. All data processing and statistical analyses were performed using SAS v9.4.

## 3 Results

### 3.1 Participating Physicians

Twenty‐three oncologists participated in this study by completing eCRFs from medical records of eligible patients. The median (quartile [Q]1–Q3) number of eligible patients across all participating physicians was 13 (7–20) and the median (Q1–Q3) number of eCRFs submitted per physician was 10 (6–10). Approximately 83% of participating oncologists self‐reported practicing in community practices and the remainder in academic medical centers or an affiliated teaching hospital. Additional physician characteristics are summarized in Table S1.

### 3.2 Patient Characteristics

A total of 181 patients who had initiated tafasitamab for R/R DLBCL were included in the analysis. One additional patient met pre‐specified eligibility criteria but was excluded from analysis as they were not administered tafasitamab as a line of therapy, but rather only as bridge therapy to CAR‐T therapy (Figure 1). Of the 181 patients included in the study, 144 (80%) were not reported as deceased during initial data collection. Of the 23 physicians contacted to provide follow‐up data on these 144 patients, 20 physicians (87%) responded and provided data on ≥ 1 patient. Follow‐up data were provided on 137 out of 144 patients (95%).

**FIGURE 1:**
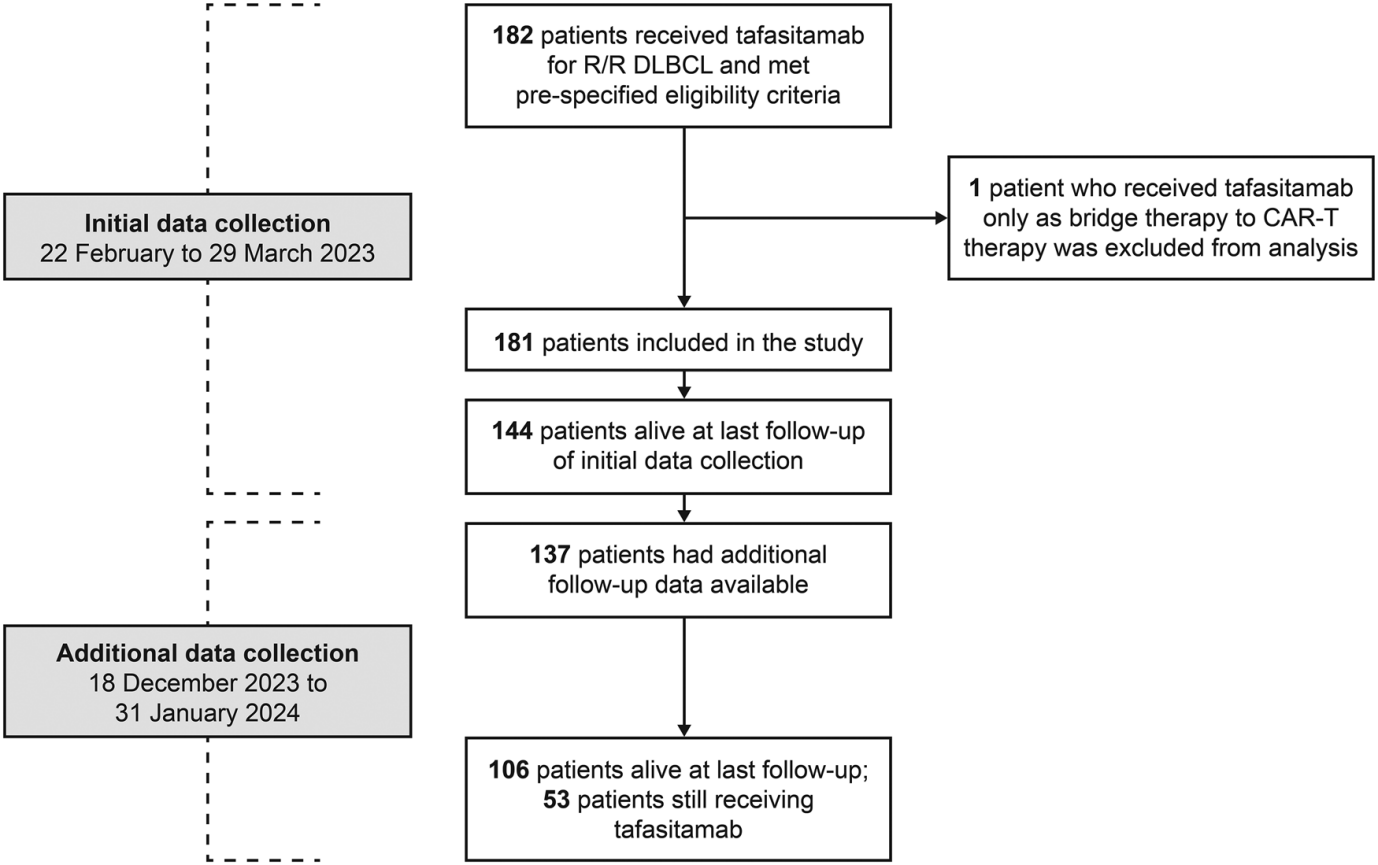
Study design. CAR‐T, chimeric antigen receptor T‐cell; DLBCL, diffuse large B‐cell lymphoma; R/R, relapsed or refractory.

Table 1 shows baseline characteristics of patients with R/R DLBCL receiving tafasitamab (*n* = 181) stratified by 2L (*n* = 130; 72%) and third‐line (3L) (*n* = 43; 24%). Eight patients (4%) received tafasitamab in fourth‐line (4L) or beyond. Median age was 71.1 years at tafasitamab initiation and 56% of patients were male. Approximately 64% of patients were White, 22% were Black, and 8% were Asian, Native Hawaiian/Pacific Islander, American Indian/Alaska Native, or mixed race. Nearly one‐fifth (17%) were of Hispanic/Latino/Latina ethnicity. At the time of tafasitamab initiation, over half had an Eastern Cooperative Oncology Group performance status (ECOG PS) of 0–1 (52%), Ann Arbor stage of III–IV (93%), and Revised International Prognostic Index (R‐IPI) score of 3–5 (80%). Forty‐seven patients (26%) had primary refractory disease. Median (Q1–Q3) study follow‐up time from tafasitamab initiation among all patients was 14.7 (8.5–17.1) months.

**TABLE 1:** Patient characteristics for overall study population and by line of treatment in which tafasitamab was received.^a^

	All patients (*N* = 181)	Tafasitamab 2L (*n* = 130)	Tafasitamab 3L (*n* = 43)
Age at tafasitamab initiation, y, median (Q1–Q3)	71.1 (65.0–75.5)	72.1 (67.2–77.1)	67.7 (62.2–73.9)
Sex at birth, male, *n* (%)	102 (56.4)	68 (52.3)	30 (69.8)
Race, *n* (%)			
White	116 (64.1)	80 (61.5)	29 (67.4)
Black/African American	40 (22.1)	31 (23.8)	9 (20.9)
Other^b^	14 (7.7)	12 (9.2)	2 (4.7)
Unknown	11 (6.1)	7 (5.4)	3 (7.0)
Ethnicity, *n* (%)			
Non‐Hispanic/Non‐Latino/Non‐Latina	149 (82.3)	109 (83.8)	35 (81.4)
Hispanic/Latino/Latina	31 (17.1)	20 (15.4)	8 (18.6)
Unknown	1 (0.6)	1 (0.8)	0
Cell of origin, *n* (%)			
GCB	81 (44.8)	60 (46.2)	17 (39.5)
Non‐GCB/ABC	39 (21.5)	28 (21.5)	9 (20.9)
Unknown	61 (33.7)	42 (32.3)	17 (39.5)
Double‐hit or triple‐hit at tafasitamab initiation, *n* (%)			
Yes, double‐/triple‐hit	22 (12.2)	14 (10.8)	8 (18.6)
Tested, found to be negative	130 (71.8)	103 (79.2)	26 (60.5)
Unknown	29 (16.0)	13 (10.0)	9 (20.9)
Ann Arbor stage at tafasitamab initiation, *n* (%)			
Stage I/II	10 (5.5)	9 (6.9)	1 (2.3)
Stage III	58 (32.0)	50 (38.5)	7 (16.3)
Stage IV	111 (61.3)	70 (53.8)	35 (81.4)
Unknown	2 (1.1)	1 (0.8)	0
ECOG PS at tafasitamab initiation, *n* (%)			
0–1	95 (52.5)	69 (53.1)	21 (48.8)
≥ 2	86 (47.5)	61 (46.9)	22 (51.2)
R‐IPI at tafasitamab initiation, *n* (% of patients with data available)^c^, ^d^			
1–2	33 (19.5)	22 (18.3)	8 (19.0)
3–5	136 (80.5)	98 (81.7)	34 (81.0)
Refractory to line before tafasitamab, *n* (%)^e^	59 (32.6)	33 (25.4)	19 (44.2)
Primary refractory, *n* (%)^f^	47 (26.0)	33 (25.4)	13 (30.2)
Primary refractory/early relapse, *n* (%)^g^	98 (54.1)	76 (58.5)	21 (48.8)
Median follow‐up time from tafasitamab initiation (Q1–Q3), mo	14.7 (8.5–17.1)	15.1 (10.6–18.2)	12.6 (7.1–15.0)

Abbreviations: 1L, first‐line; 2L, second‐line; 3L, third‐line; 4L, fourth‐line; 5L, fifth‐line; ABC, activated B cell; ECOG PS, Eastern Cooperative Oncology Group performance status; GCB, germinal centre B cell; Q1, first quartile; Q3, third quartile; R‐IPI, Revised International Prognostic Index.

^a^ Although some patients received tafasitamab as 4L or 5L therapy, data for these patients are not shown separately due to small numbers.

^b^ Asian, Native Hawaiian/Pacific Islanders, American Indian/Alaska Native, or mixed race.

^c^ 169 patients had data available to calculate R‐IPI score; R‐IPI score was calculated based on individual data elements, and the following variables were assigned an incremental point score according to the degree of elevation: stage (Ann Arbor), age, ECOG PS, extranodal site(s), and lactate dehydrogenase [25].

^d^ There were no patients with an R‐IPI score of 0 (very good prognosis) at tafasitamab initiation.

^e^ Defined as disease progression while receiving line of therapy before tafasitamab or disease progression occurring ≤ 6 months after completion of the line of therapy before tafasitamab.

^f^ Defined as disease progression while receiving 1L therapy or disease progression occurring ≤ 6 months after completion of 1L therapy.

^g^ Defined as disease progression while receiving 1L therapy or disease progression occurring ≤ 12 months after completion of 1L therapy.

### 3.3 Tafasitamab‐Based Regimen Utilisation Patterns

Tafasitamab‐based regimen utilisation patterns are shown in Table 2. The majority of patients (97%) had a tafasitamab starting dose of 12 mg/kg, with the remaining five patients having a starting dose of 6 mg/kg. Median (Q1–Q3) time from initial diagnosis of DLBCL to tafasitamab initiation was 20.0 (12.5–34.7) months.

**TABLE 2:** Tafasitamab regimen utilisation patterns.

Variable	All patients (*N* = 181)
DLBCL line of therapy in which tafasitamab was received, *n* (%)	
2L	130 (71.8)
3L	43 (23.8)
4L	5 (2.8)
5L	3 (1.7)
Starting dose of tafasitamab, *n* (%)	
12 mg/kg	176 (97.2)
6 mg/kg	5 (2.8)
Prior ASCT therapy, *n* (%)	21 (11.6)
Prior CAR‐T therapy, *n* (%)	6 (3.3)
Subsequent CAR‐T therapy, *n* (%)	9 (5.0)
Concomitant lenalidomide, *n* (%)	174 (96.1)
Lenalidomide starting strength, *n* (%)	
25 mg	68 (37.6)
20 mg	44 (24.3)
15 mg	29 (16.0)
10 mg	31 (17.1)
5 mg	2 (1.1)
No concomitant lenalidomide	7 (3.9)
Tafasitamab discontinued at most recent follow‐up, *n* (%)	128 (70.7)
Tafasitamab TTD, mo, median (95% CI)^a^	11.0 (9.4–12.2)

Abbreviations: 2L, second‐line; 3L, third‐line; 4L, fourth‐line; 5L, fifth‐line; ASCT, autologous stem cell transplant; CAR‐T, chimeric antigen receptor T‐cell; CI, confidence interval; DLBCL, diffuse large B‐cell lymphoma; TTD, time to treatment discontinuation.

^a^ Difference between the date of discontinuation for any reason and the date of initiation; patients still on therapy at data collection were censored at last follow‐up date.

Most patients (*n* = 174; 96%) received lenalidomide with tafasitamab treatment; the most frequently administered strength of lenalidomide was 25 mg (among 38%) (Table 2). The most frequently cited lenalidomide dosing schedule was daily for 21 days, repeating every 28 days (among 97%).

Among those who received concomitant lenalidomide, 35 patients (20%) had ≥ 1 lenalidomide dose reduction during treatment (data not shown). The most common reasons for lenalidomide dose reductions were neutropenia (74%), thrombocytopenia (31%), performance status/patient frailty (29%), and renal dysfunction (20%) (not mutually exclusive; hence, total is > 100%).

At the time of most recent follow‐up, 128 patients (71%) had discontinued tafasitamab (Table 2). Median (95% CI) time to treatment discontinuation for tafasitamab was 11.0 months (9.4–12.2). Among the 128 patients who discontinued tafasitamab, reasons for discontinuation included progression confirmed by scan (62%), progression defined clinically (9%), toxicity (10%), patient/caregiver request (7%), CR (4%), and other reasons (8%) (data not shown).

### 3.4 Overall Treatment Patterns and Treatment Sequencing

The treatment journey of patients, including treatments before tafasitamab initiation, is shown in Figure S1. The majority (*n* = 159; 88%) of patients received rituximab with cyclophosphamide, doxorubicin, vincristine, and prednisone (R‐CHOP) as 1L therapy for DLBCL. The most common 2L therapy was tafasitamab (*n* = 130; 72%). Before tafasitamab treatment, 21 patients (12%) received ASCT therapy (as 2L). Among patients who did not receive ASCT as 2L therapy before tafasitamab, the most frequently cited reasons for not receiving ASCT were patient refusal, not otherwise specified (23%), other organ dysfunction or comorbidities precluding use of ASCT on the basis of unacceptable risk of treatment (21%), and left ventricular ejection fraction < 50% by echocardiogram (19%) (data not shown). Six patients (3%) received CAR‐T therapy (as 2L or 3L) before tafasitamab. Nine patients (5%) received CAR‐T therapy after treatment with tafasitamab, all of whom received their CAR‐T therapy in the line of therapy immediately after tafasitamab.

### 3.5 Effectiveness Outcomes

Best response to tafasitamab therapy was available for 170 patients (94%) and is shown for the overall population and by treatment line in Figure S2. Among these 170 patients, the criteria used for evaluating best response included Lugano [18] (*n* = 124; 73%), Cheson 2007 [19] (*n* = 45; 26%), and other (*n* = 1; 1%) (data not shown). Among the overall study population, real‐world overall response rate (ORR) (95% CI) for tafasitamab was 73.5% (*n* = 133; 67.0–79.9), whereas real‐world CR (rwCR) rate (95% CI) was 23.2% (*n* = 42; 17.1–29.4) and real‐world partial response (rwPR) rate was 50.3% (*n* = 91; 43.0‐57.6). Among patients who achieved a rwCR or rwPR, median (95% CI) duration of response (mDOR) was 9.6 (8.3–13.3) months. mDOR (95% CI) was 19.2 (8.8–not evaluable [NE]) months among those achieving a rwCR and 8.5 (6.8–10.0) months among those with a rwPR as best response (Figure 2).

**FIGURE 2:**
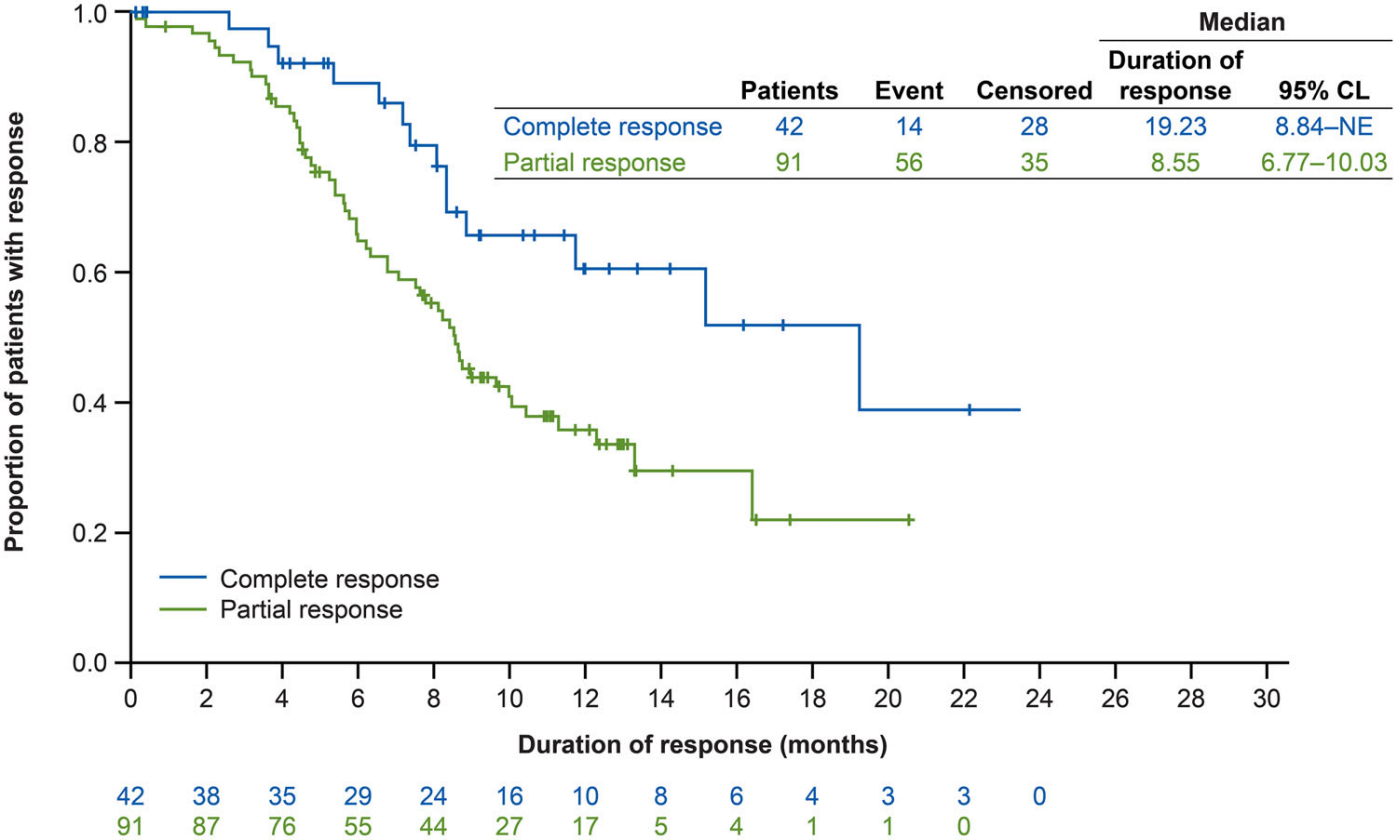
Duration of response^a^ among those who experienced CR or PR as a best response to tafasitamab. ^a^Duration of response was calculated as the time from when CR or PR was first observed to the time of physician‐reported disease progression or death from any cause, whichever occurred first. For patients with no documented progression event, a progression event was imputed at date of discontinuation if reason for discontinuation was reported as disease progression. Patients with no documented progression event were censored at start date of new anticancer therapy if reason for discontinuation was not reported as disease progression. Patients with no documented progression event or start date of new anticancer therapy were censored at date of discontinuation or at date of last encounter if patient was still receiving tafasitamab. CL, confidence limit; CR, complete response; NE, not evaluable; PR, partial response.

Additional effectiveness measures (unadjusted) are provided for the overall study population and for pre‐specified subgroups of clinical interest in Table S2. Median (95% CI) rwPFS and rwOS for the overall study population were 11.3 (9.8–13.6) months and 24.8 (17.8–NE) months, respectively.

The variables listed in Table S3 were included in univariable analyses to assess their potential association with rwPFS and, separately, rwOS. The final multivariable Cox model identified the following factors associated (*p *< 0.05) with increased risk of progression when adjusted for other patient and treatment characteristics: tafasitamab in 3L–5L (fifth line; vs. 2L; hazard ratio [HR], 1.79; 95% CI, 1.20–2.66; *p *= 0.004), increasing National Cancer Institute Charlson Comorbidity Index scores (included in model as a continuous variable; HR, 1.43; 95% CI, 1.03–1.97; *p *= 0.033), and bulky disease (vs. non‐bulky disease; HR, 1.96; 95% CI, 1.26–3.05; *p *= 0.003) (Table 3). Ann Arbor Stage I–II (vs. stage III–IV; HR, 0.30; 95% CI, 0.10–0.95; *p *= 0.041) was associated with a lower risk of progression. The proportional hazards assumption was tested for all covariates. The assumption was violated for the ECOG PS ≥ 2 versus < 2 variable, indicating that the effect of ECOG PS on progression was not constant over time. To address this violation, we employed a stratified Cox proportional hazards model, stratifying by ECOG PS (≥ 2 vs. < 2). This approach allows for different baseline hazard functions for each stratum, thereby accommodating the non‐proportionality of the ECOG PS variable. Figure S3 illustrates the survival function for an average patient when stratified by ECOG PS ≥ 2 versus < 2. The figure indicates that overall, patients with ECOG PS ≥ 2 have a higher risk of progression than patients with ECOG PS < 2; however, this difference is not consistent over time.

**TABLE 3:** Factors associated with rwPFS and rwOS among patients who initiated tafasitamab for R/R DLBCL.

Characteristic	*N*	Hazard ratio (95% CI)	*p* value
**Final Cox model of factors associated with rwPFS among patients initiating tafasitamab for R/R DLBCL** ^a^			
Line of therapy in which patient received tafasitamab			
3L–5L	51	1.79 (1.20–2.66)	0.004
2L (reference)	130	—	—
Ann Arbor stage at initiation of tafasitamab			
Stage I–II	10	0.30 (0.10–0.95)	0.041
Stage III–IV (reference)	169	—	—
Charlson Comorbidity Index score (National Cancer Institute version) at initiation of tafasitamab (continuous)	181	1.43 (1.03–1.97)	0.033
Tumour volume at initiation of tafasitamab			
Bulky	36	1.96 (1.26–3.05)	0.003
Non‐bulky	145	—	—
**Final Cox model of factors associated with rwOS among patients initiating tafasitamab for R/R DLBCL**			
Age at tafasitamab initiation, years (continuous)	181	1.06 (1.03–1.09)	< 0.001
Line of therapy in which patient received tafasitamab			
3L–5L	51	2.39 (1.46–3.93)	< 0.001
2L (reference)	130	—	—
ECOG PS at initiation of tafasitamab			
≥ 2	86	3.57 (2.13–5.97)	< 0.001
< 2 (reference)	95	—	—
Tumour volume at initiation of tafasitamab			
Bulky	36	2.22 (1.27–3.87)	0.005
Non‐bulky (reference)	145	—	—

Abbreviations: 2L, second‐line; 3L, third‐line; 5L, fifth‐line; CI, confidence interval; DLBCL, diffuse large B‐cell lymphoma; ECOG PS, Eastern Cooperative Oncology Group performance status; OS, overall survival; PFS, progression‐free survival; R/R, relapsed or refractory; rw, real‐world.

^a^ To overcome violation of proportional hazards assumption caused by the variable ECOG PS, coefficients were adjusted for ECOG PS ≥ 2 versus < 2 by stratifying the model on ECOG PS.

When adjusted for other patient and treatment characteristics, a Cox proportional hazards model found the following factors associated with increased mortality (Table 3): increasing age (included in model as a continuous variable; HR, 1.06; 95% CI, 1.03–1.09; *p *< 0.001), tafasitamab in 3L–5L (vs. 2L; HR, 2.39; 95% CI, 1.46–3.93; *p *< 0.001), ECOG PS ≥ 2 (vs. < 2; HR, 3.57; 95% CI, 2.13–5.97; *p* < 0.001), and bulky disease (vs. non‐bulky disease; HR, 2.22; 95% CI, 1.27–3.87; *p *= 0.005).

## 4 Discussion

This real‐world study describes characteristics, treatment patterns, and outcomes of a population of 181 US patients who received tafasitamab for R/R DLBCL since October 21, 2020. Importantly, this study describes tafasitamab outcomes and utilisation among a large number of patients from both community and academic settings and adds to the currently limited knowledge base. Although larger real‐world tafasitamab investigations have examined pharmacovigilance [17], available data on the use and effectiveness of tafasitamab in the US real‐world setting currently include a limited number of patients, are predominantly from academic centers, or focus on certain treatment sequences [11, 12, 14, 15, 16].

The patient population of this study was racially and ethnically diverse, reflecting the diversity of the larger US DLBCL population; nearly one‐third were from typically underrepresented racial minority groups and approximately one‐sixth were of Hispanic ethnicity. Approximately one‐fifth of US adults diagnosed with DLBCL are non‐White [20]. However, racial minority populations are typically underrepresented in DLBCL clinical trials (for example, 89% of the L‐MIND patient population was White) [8] and in some settings (for example, in a recently published real‐world study across academic practice centers, 90% of the patient population was White) [13]. Hence, the real‐world evidence generated in this study helps to bridge the knowledge gap regarding tafasitamab treatment utilisation and clinical outcomes in typically underrepresented populations.

In comparison with the population enrolled in the pivotal L‐MIND trial [8], this study included a higher proportion of patients with primary refractory disease (26% vs. 19%), patients who received tafasitamab as 2L therapy (72% vs. 50%), and patients with double‐ or triple‐hit disease (12% vs. 2%) [9]. Nearly all patients in this study population had an ECOG PS of 0–2 (in alignment with the L‐MIND clinical trial ECOG PS criteria). However, compared with the L‐MIND population, there was a higher proportion of patients in this study with poor prognostic indicators, including an Ann Arbor stage of III–IV (93% vs. 75%), and R‐IPI/IPI score of 3–5 (80% vs. 51%), although R‐IPI/IPI was not calculable for all patients in this study [8].

ORR was higher in the current study (73%) versus that reported in L‐MIND (60%) [8]. Drivers underlying this difference are currently unknown; however, differences in patient and disease characteristics (described previously), as well as varying response assessment criteria, modalities, and frequency between clinical trials and real‐world settings, should be considered [21]. In this study, response was determined by the physician abstractor, and the response criteria used for assessment was at the physician's discretion, which may have contributed to higher variability and/or bias in response assessments among patients in this study.

Differences in baseline characteristics, treatment patterns, and tafasitamab‐associated outcomes were observed between this study and a recently published real‐world study of similar size examining tafasitamab use in the United States (Qualls et al.) [13]. In comparison with the Qualls et al. study, the patient population in this study was more diverse (64% vs. 90% White) and had a lower proportion with primary refractory disease (26% vs. 49%) [13]. In addition, patients in this study received tafasitamab earlier in their course of therapy, with 72% versus 28% receiving tafasitamab as 2L therapy and with < 5% versus 38% receiving tafasitamab as 4L+ therapy in this study versus the Qualls et al. study, respectively [13]. The majority of patients in this study were treated in community practice settings, whereas patients in the Qualls et al. study were treated in academic settings. The differences in baseline characteristics and treatment patterns in each study may reflect differences in clinical practice between community and academic centers. Compared with the Qualls et al. study findings, patients included in this study had higher ORR (73% vs. 31%) [13], which is likely related, at least in part, to some of the aforementioned differences in baseline characteristics and line of therapy in which tafasitamab was received.

Optimal sequencing of novel therapies for treatment of DLBCL has not been established, especially as it relates to sequential use of therapies targeting CD19 (for example, CAR‐T agents, tafasitamab) [22, 23]. In the patient population included in this real‐world study, most received R‐CHOP as 1L therapy and then went on to receive tafasitamab (the majority of whom received tafasitamab in combination with lenalidomide) as 2L therapy. Notably, in the present study, multivariable Cox modelling for rwPFS and rwOS indicated that tafasitamab used as 2L therapy (vs. 3L–5L) was associated with longer PFS and OS, which is consistent with findings from L‐MIND, which reported longer median PFS and OS in patients treated with tafasitamab as 2L versus later lines of therapy [8, 24]. Based on these findings, consideration of the utilisation of tafasitamab with lenalidomide in earlier lines of therapy (2L or 3L) for appropriate patients with DLBCL is a reasonable therapeutic approach.

In summary, the majority of patients initiating tafasitamab for R/R DLBCL in this real‐world study received tafasitamab in combination with lenalidomide as 2L therapy, at a dosage consistent with labelling (12 mg/kg) [7]. In addition, these results demonstrate clinical benefit of tafasitamab in this real‐world setting and indicate lower risk of progression and mortality associated with earlier use of tafasitamab (2L vs. 3L–5L).

### 4.1 Study Limitations

As with any real‐world study, this study may be limited by unobserved data and missing data. Source document verification was not conducted; however, all physicians were required to submit to data validation checks. Most physicians abstracted data for nearly the entire eligible cohort at their practice (median number of eligible patients was 13), minimising the potential for selection bias. However, the study included data abstracted by a limited number of oncologists (*N* = 23), and treatment patterns and outcomes may not be reflective of the broader population of oncologists managing patients with DLBCL in the United States. Findings from this study may be impacted by a lack of uniform assessment criteria for certain variables, such as disease response.

## 5 Conclusion

Our real‐world analysis expands the limited existing evidence base on the effectiveness of tafasitamab in a diverse population of patients with R/R DLBCL treated in community and academic settings in the United States. Multivariable models of rwPFS and rwOS suggested that 2L use of tafasitamab provided the most benefit, compared with use as later lines of therapy (3L–5L). These data support the clinical efficacy of tafasitamab when used in early lines of treatment of R/R DLBCL, as demonstrated in the L‐MIND trial.

## Author Contributions

K.S., T.A., J.G., B.F., and L.J.N. designed the study. K.S., T.A., J.G., B.F., P.P., L.J.N., and N.E. were responsible for data collection. P.P. and P.A. analysed the data. All authors contributed to the interpretation of the data. P.P. and P.A. had full access to all the data in the study and took responsibility for the integrity of the data and the accuracy of the data analysis. All authors contributed to the drafting and critical review of the manuscript and approved the final draft for submission.

## Ethics Statement

The study followed all standard research guidelines and received approval by a central institutional review board.

## Consent

The study received exemption for obtaining patient informed consent by a central institutional review board.

## Conflicts of Interest

Kim Saverno, John Galvin, and Theresa Amoloja report employment by and stock ownership in Incyte Corporation. Loretta J. Nastoupil reports honorarium from AbbVie, AstraZeneca, BeiGene, Bristol Myers Squibb, Caribou Biosciences, Daiichi Sankyo, Genentech/Roche, Genmab, Gilead Sciences/Kite Pharma, Incyte Corporation, Ipsen, Janssen, Merck, Novartis, Regeneron, and Takeda; and research support from BeiGene, Bristol Myers Squibb, Caribou Biosciences, Daiichi Sankyo, Genentech, Genmab, Gilead Sciences/Kite Pharma, Janssen, Merck, Novartis, and Takeda. Bruce Feinberg and Danielle Gentile report employment by and stock ownership in Cardinal Health. Prathamesh Pathak and Parisa Asgarisabet report employment by Cardinal Health. Narendranath Epperla reports research funding (to the institution) from BeiGene, Incyte Corporation, ADC Therapeutics, Ipsen, and Lilly; Advisory board member for CRISPR Therapeutics, Genentech, and Ipsen.

## Supporting information




**Supporting File 1**: jha270256‐sup‐0001‐figureS1.tif

**Supporting File 2**: jha270256‐sup‐0002‐figureS2.tif

**Supporting File 3**: jha270256‐sup‐0003‐figureS3.tif

**Supporting File 4**: jha270256‐sup‐0004‐SuppMat.docx

## Data Availability

De‐identified data are not available for this retrospective real‐world study. For other data‐related queries, please contact the corresponding author, Kim Saverno (ksaverno@incyte.com).
