# Supplementary material for: A Real‐World Study of Tafasitamab for the Treatment of Relapsed or Refractory Diffuse Large B‐Cell Lymphoma in the United States
Source: EJHaem. 2026 Mar 26;7(2):e70256. doi: 10.1002/jha2.70256 (PMC13629462; doi:10.1002/jha2.70256)
Supplement: Supplementary file 1 [file JHA2-7-e70256-s004.pdf]

SUPPORTING MATERIAL

A real-world study of tafasitamab for the treatment of relapsed or refractory diffuse large B-cell lymphoma in the United States

Kim Saverno, Loretta J. Nastoupil, Bruce Feinberg, John Galvin, Prathamesh Pathak, Theresa Amoloja, Danielle Gentile, Parisa Asgarisabet, Narendranath Epperla

Supplemental Table 1. Physician characteristics
Supplemental Table 2. Outcomes (unadjusted) among overall study population and pre-specified subgroups of interest
Supplemental Table 3. Factors evaluated for their association with rwPFS and rwOS
Supplemental Figure 1. Sankey plot depicting patient treatment journey
Supplemental Figure 2. Real-world best response rates
Supplemental Figure 3. Illustration of rwPFS curve when Cox model is stratified by ECOG PS to overcome proportional hazards violation

Supplemental Tables

SUPPLEMENTAL TABLE 1 Physician characteristics

	**All providers (*N* = 23)**
Primary practice setting, *n* (%)	
Solo practitioner	0
Small private community practice (2–5 physicians)	4 (17.4)
Small private community practice (2–5 physicians) owned by hospital	1 (4.4)
Medium private community practice (6–10 physicians)	5 (21.7)
Medium private community practice (6–10 physicians) owned by hospital	1 (4.4)
Large private community practice (>10 physicians)	8 (34.8)
Academic medical centre	2 (8.7)
Affiliated teaching hospital	2 (8.7)
Provider number of years in practice, *n* (%)	
Mean (SD)	16.7 (6.3)
Median (Q1–Q3)	16 (11.0-20.0)
Practice urbanicity, *n* (%)[a]	
Urban	11 (47.8)
Suburban	9 (39.1)
Rural	3 (13.0)
US geographic location, *n* (%)[b]	
South	9 (39.1)
Northeast	5 (21.7)
West	5 (21.7)
Midwest	4 (17.4)
Medical specialty, *n* (%)[c]	
Medical oncology	5 (21.7)
Haematology/oncology	22 (95.7)

Q1, first quartile; Q3, third quartile.
[a] Definitions of urban, suburban, or rural were not provided to physicians.
[b] Midwest includes Illinois, Indiana, Iowa, Kansas, Michigan, Minnesota, Missouri, Nebraska, North Dakota, Ohio, South Dakota, and Wisconsin; Northeast includes Connecticut, Delaware, Maine, Maryland, Massachusetts, New Hampshire, New Jersey, New York, Pennsylvania, Rhode Island, and Vermont; South includes Alabama, Arkansas, District of Columbia, Florida, Georgia, Kentucky, Louisiana, Mississippi, North Carolina, Oklahoma, South Carolina, Tennessee, Texas, Virginia, and West Virginia; West includes Alaska, Arizona, California, Colorado, Hawaii, Idaho, Montana, Nevada, New Mexico, Oregon, Utah, Washington, and Wyoming.
[c] Categories not mutually exclusive.

SUPPLEMENTAL TABLE 2 Outcomes (unadjusted) among overall study population and pre-specified subgroups of interest

	**Effectiveness measures**			
	**rwPFS,[a] median (95% CI), months**	**rwOS,[b] median (95% CI), months**	**rwDOR,[c] median (95% CI), months**	**rwORR,[d] (95% CI), %**
**Overall study population**				
Overall (*N* = 181)	11.3 (9.8–13.6)	24.8 (17.8–NE)	9.6 (8.3–13.3)	73.5 (67.0–79.9)
**Subgroups of interest**				
Line of therapy				
2L (*n* = 130)	13.2 (10.6–16.4)	NR (19.2–NE)	10.4 (8.5–16.4)	78.5 (71.4–85.5)
3L (*n* = 43)	9.0 (6.8–12.0)	13.6 (9.1–NE)	8.1 (4.9–12.3)	62.8 (48.3–77.2)
Primary refractory[e]				
Yes (*n* = 47)	9.8 (6.8–13.8)	NR (13.0–NE)	8.5 (6.0–NE)	66.0 (52.4–79.5)
No (*n* = 134)	11.7 (10.0–15.3)	24.8 (17.8–NE)	10.0 (8.3–15.2)	76.1 (68.9–83.3)
Bulky disease				
Yes (*n* = 36)	10.1 (6.1–12.8)	14.8 (9.3–NE)	7.1 (4.9–8.7)	63.9 (47.0–80.8)
No (*n* = 145)	11.9 (9.8–15.3)	NR (18.3–NE)	10.4 (8.5–15.2)	75.9 (68.7–83.0)

1L, first-line; 2L, second-line; 3L, third-line; CI, confidence interval; CR, complete response; DOR, duration of response; NE, not evaluable; NR, not reached; ORR, overall response rate; OS, overall survival; PFS, progression-free survival; PR, partial response; rw, real-world.

[a] rwPFS was defined as the time from tafasitamab initiation to disease progression or death from any cause, whichever occurred first.

[b] Time from tafasitamab initiation to death from any cause.

[c] Among patients for whom a CR or PR was observed during tafasitamab treatment, DOR was calculated as the time from when CR or PR was first observed to the time of physician-reported disease progression or death from any cause, whichever occurred first.

[d] Sum of patients with reported rwCR and patients with rwPR as their reported best response to tafasitamab divided by the total number of patients in the cohort and reported as a percentage.

[e] Defined as disease progression while receiving 1L therapy or disease progression occurring ≤6 months after completion of 1L therapy.

SUPPLEMENTAL TABLE 3 Factors evaluated for their association with rwPFS and rwOS

Treating physician practice setting
Academic
Non-academic (reference)
Age at tafasitamab initiation, years (continuous)
Sex at birth
Female
Male (reference)
Race
African American
Other + unknown
White (reference)
Ethnicity
Hispanic
Non-Hispanic + unknown (reference)
Most recent primary insurance/payer at data collection
Non-Medicare + unknown
Medicare (reference)
US region of residence[a]
Midwest
Northeast
West
South (reference)
Line of therapy in which patient received tafasitamab, 3L–5L (reference, 2L)
3L–5L
2L (reference)
Patient was deemed primary refractory[b]
Yes
No (reference)
Patient was deemed primary refractory or early relapse[c]
Yes
No (reference)
Ann Arbor stage at initiation of tafasitamab
Stage I–II
Stage III–IV (reference)
ECOG PS at initiation of tafasitamab
≥2
<2 (reference)
Extranodal site involvement at initiation of tafasitamab
Yes
No (reference)
Serum lactate dehydrogenase level at initiation of tafasitamab
<1 (normal)
>1 (abnormal) (reference)
Charlson Comorbidity Index score (National Cancer Institute version) at initiation of tafasitamab (evaluated both as a continuous and categorical variable; categories listed below)
0–1
>1
0 (reference)
Presence of double-hit or triple-hit B-cell lymphoma at initiation of tafasitamab
Yes
No (reference)
Tumour volume at initiation of tafasitamab

Bulky	
Non-bulky (reference)	

2L, second-line; 3L, third-line; 5L, fifth-line; ECOG PS, Eastern Cooperative Oncology Group performance status; OS, overall survival; PFS, progression-free survival; rw, real-world.

[a] Midwest includes Illinois, Indiana, Iowa, Kansas, Michigan, Minnesota, Missouri, Nebraska, North Dakota, Ohio, South Dakota, and Wisconsin; Northeast includes Connecticut, Delaware, Maine, Maryland, Massachusetts, New Hampshire, New Jersey, New York, Pennsylvania, Rhode Island, and Vermont; South includes Alabama, Arkansas, District of Columbia, Florida, Georgia, Kentucky, Louisiana, Mississippi, North Carolina, Oklahoma, South Carolina, Tennessee, Texas, Virginia, and West Virginia; West includes Alaska, Arizona, California, Colorado, Hawaii, Idaho, Montana, Nevada, New Mexico, Oregon, Utah, Washington, and Wyoming.

[b] Defined as disease progression while receiving first-line therapy or disease progression occurring ≤6 months after completion of first-line therapy.

[c] Defined as disease progression while receiving first-line therapy or disease progression occurring within ≤12 months after completion of first-line therapy.

Supplemental Figures

SUPPLEMENTAL FIGURE 1 Sankey plot depicting patient treatment journey. Bridge therapy followed by CAR-T was classified as a single line of therapy and salvage chemotherapy followed by ASCT was classified as a single line of therapy. 1L, first-line; 2L, second-line; 3L, third-line; 4L, fourth-line; 5L, fifth-line; ASCT, autologous stem cell transplant; B, bendamustine; CAR-T, chimeric antigen receptor T-cell therapy; len, lenalidomide; pola, polatuzumab; R-CHOP, rituximab with cyclophosphamide, doxorubicin, vincristine, and prednisone; R, rituximab; tafa, tafasitamab.

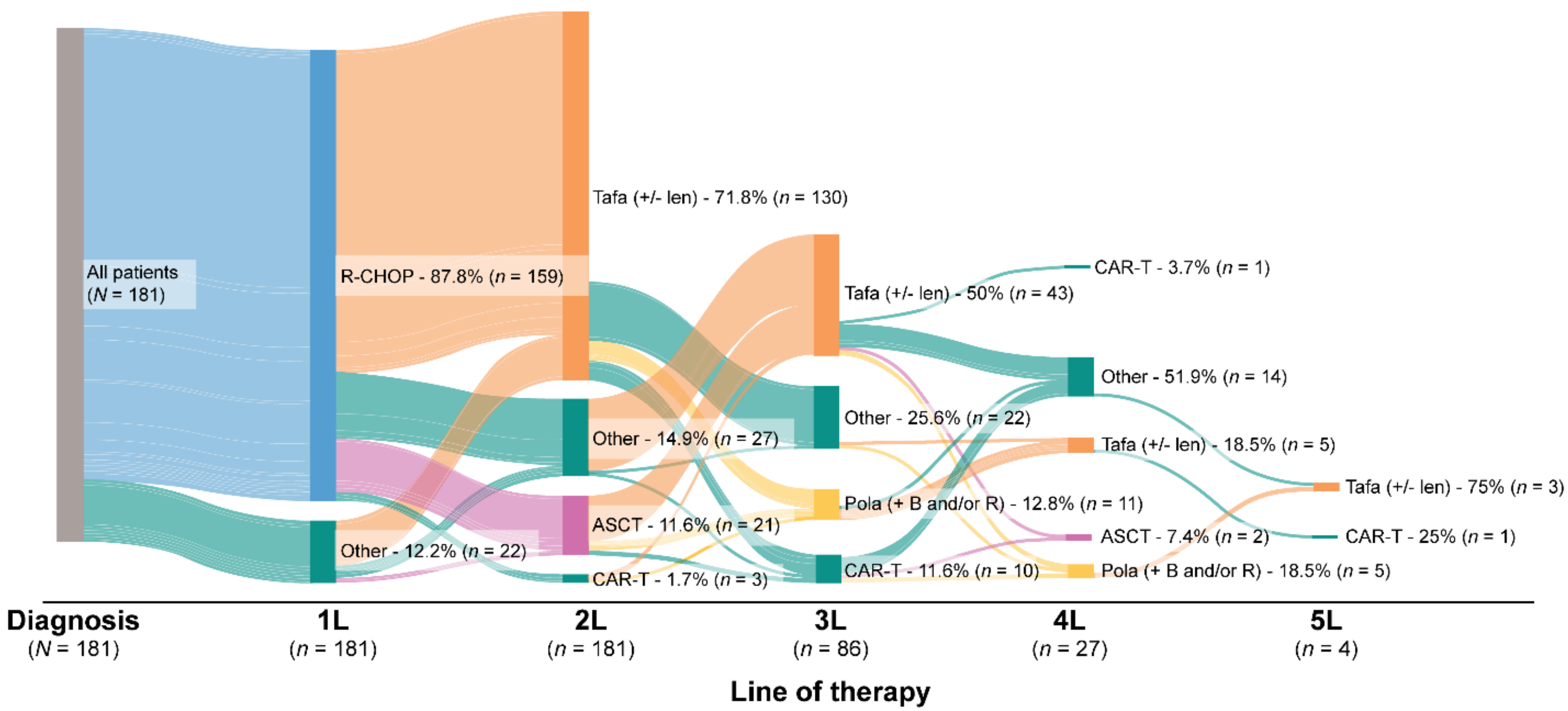

SUPPLEMENTAL FIGURE 2 Real-world best response rates. 2L, second-line; 3L, third-line; CR, complete response; ORR, overall response rate; PD, progressive disease; PR, partial response; rw, real-world; SD, stable disease.

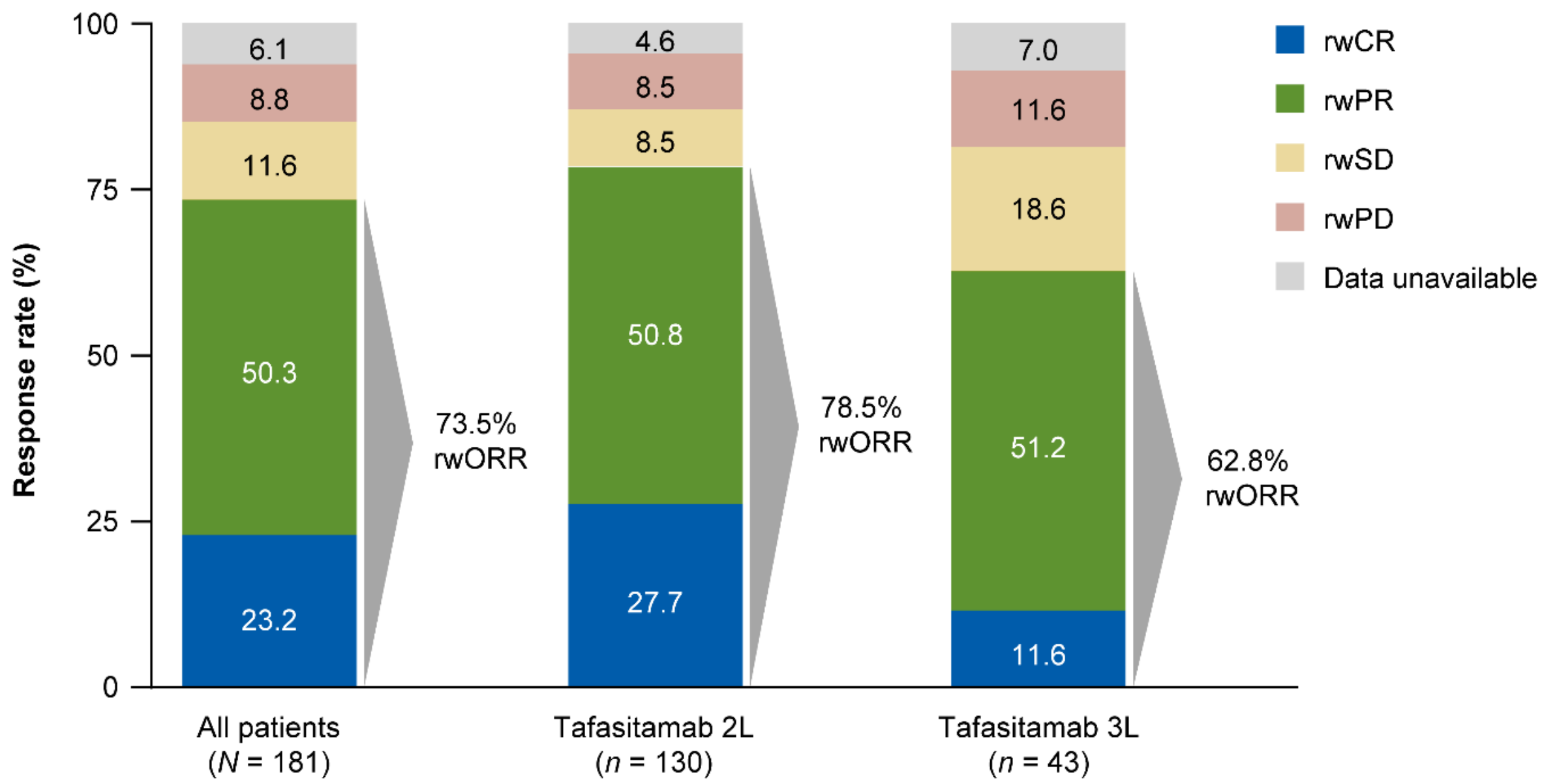

SUPPLEMENTAL FIGURE 3 Illustration of rwPFS curve when Cox model is stratified by ECOG PS to overcome proportional hazards violation. Survivor functions with 95% CIs are shown. As illustrated, patients with an ECOG PS of <2 have improved PFS vs. those with an ECOG PS score of ≥2; however, the survival curves are not proportional over time. 2L, second-line; CI, confidence interval; ECOG PS, Eastern Cooperative Oncology Group performance status; NCI, National Cancer Institute; PFS, progression-free survival; rw, real-world.

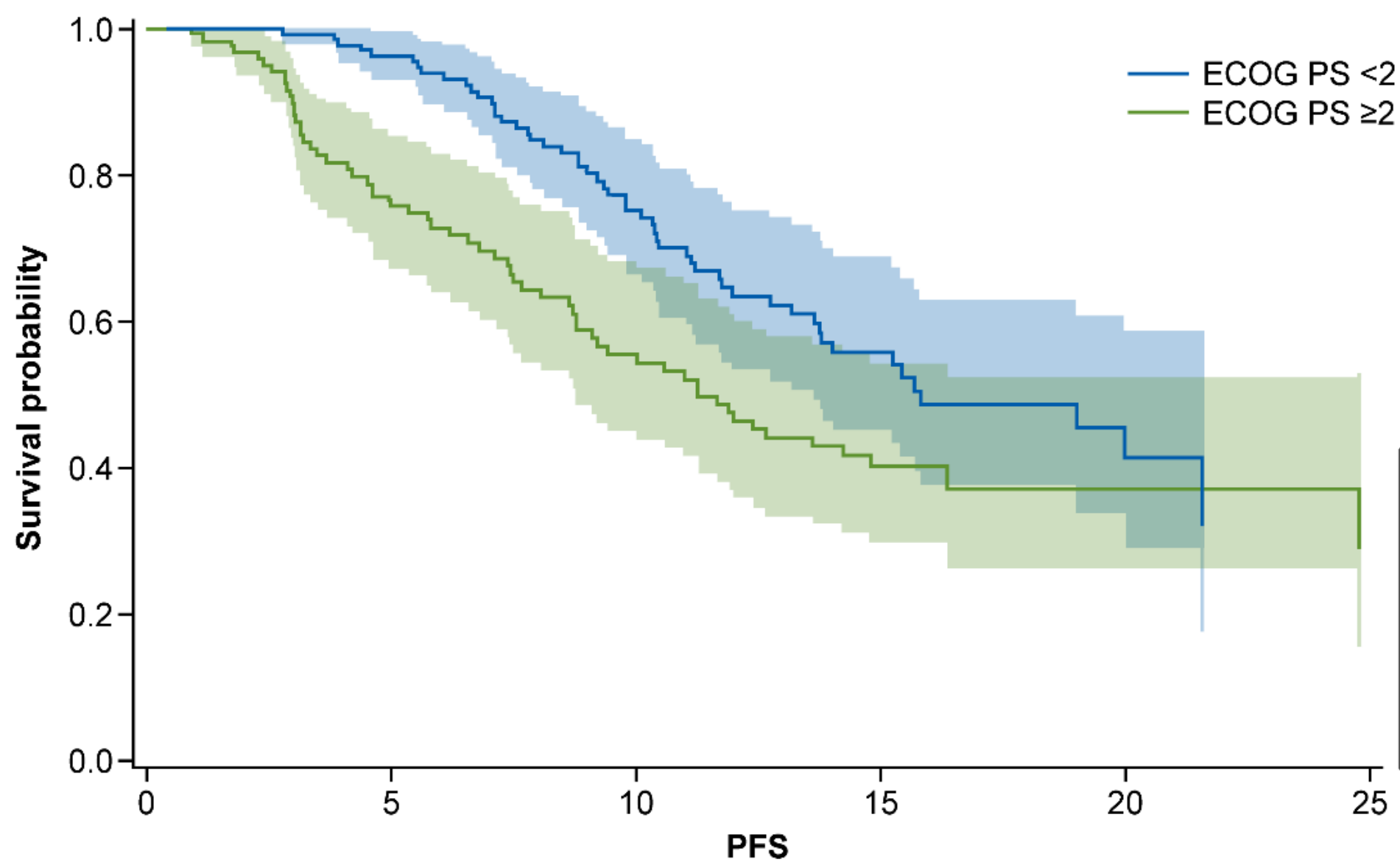

Characteristics of patients in Cox model, on average	
**Line of therapy**	2L
**Stage**	Stage III–IV
**Tumor volume**	Non-bulky
**NCI Charlson Comorbidity Index**	0.484
